# The Crosstalk of Endoplasmic Reticulum (ER) Stress Pathways with NF-κB: Complex Mechanisms Relevant for Cancer, Inflammation and Infection

**DOI:** 10.3390/biomedicines6020058

**Published:** 2018-05-16

**Authors:** M. Lienhard Schmitz, M. Samer Shaban, B. Vincent Albert, Anke Gökçen, Michael Kracht

**Affiliations:** 1 Institute of Biochemistry, Justus Liebig University Giessen, D-35392 Giessen, Germany; lienhard.schmitz@biochemie.med.uni-giessen.de; 2 Rudolf-Buchheim-Institute of Pharmacology, Justus Liebig University Giessen, D-35392 Giessen, Germany; Mohammed.S.Shaban@pharma.med.uni-giessen.de (M.S.S.); Benadict.V.Albert@pharma.med.uni-giessen.de (B.V.A.); Anke.Goekcen@pharma.med.uni-giessen.de (A.G.); 3 Rudolf-Buchheim-Institute of Pharmacology, Universities of Giessen and Marburg Lung Center (UGMLC), Schubertstrasse 81, D-35392 Giessen, Germany

**Keywords:** ER stress, cancer, infection, inflammation, unfolded protein response, NF-κB, IκBα, thapsigargin

## Abstract

Stressful conditions occuring during cancer, inflammation or infection activate adaptive responses that are controlled by the unfolded protein response (UPR) and the nuclear factor of kappa light polypeptide gene enhancer in B-cells (NF-κB) signaling pathway. These systems can be triggered by chemical compounds but also by cytokines, toll-like receptor ligands, nucleic acids, lipids, bacteria and viruses. Despite representing unique signaling cascades, new data indicate that the UPR and NF-κB pathways converge within the nucleus through ten major transcription factors (TFs), namely activating transcription factor (ATF)4, ATF3, CCAAT/enhancer-binding protein (CEBP) homologous protein (CHOP), X-box-binding protein (XBP)1, ATF6α and the five NF-κB subunits. The combinatorial occupancy of numerous genomic regions (enhancers and promoters) coordinates the transcriptional activation or repression of hundreds of genes that collectively determine the balance between metabolic and inflammatory phenotypes and the extent of apoptosis and autophagy or repair of cell damage and survival. Here, we also discuss results from genetic experiments and chemical activators of endoplasmic reticulum (ER) stress that suggest a link to the cytosolic inhibitor of NF-κB (IκB)α degradation pathway. These data show that the UPR affects this major control point of NF-κB activation through several mechanisms. Taken together, available evidence indicates that the UPR and NF-κB interact at multiple levels. This crosstalk provides ample opportunities to fine-tune cellular stress responses and could also be exploited therapeutically in the future.

## 1. Introduction

The NF-κB pathways regulate the activities of a family of five transcription factors (RELA (p65), RELB, c-REL, NFKB1 (p105/p50) and NFKB2 (p100/p52)) that play numerous roles in physiological, but also pathophysiological, conditions [1]. The pivotal role of NF-κB in promoting several of the ten hallmarks of cancer is well established and has been the subject of excellent reviews [2,3,4].

NF-κB is an inducible transcription factor that displays low constitutive background activity. It can be strongly induced by a variety of agents which increase the nuclear concentration of the DNA-binding subunits and subsequently, promote the binding to specific cognate κB sites across the genome. The NF-κB system acts as a general stress sensor that is activated by a multitude of adverse conditions. These are extracellular activators, such as infection or inflammation, but also intracellular activators, such as DNA damage or the unfolded protein response (UPR). Here, we discuss recent progress in the analysis of (reciprocal) interactions between the NF-κB pathways and the UPR, a pathway that controls the folding capacities of the endoplasmic reticulum (reviewed in [5].

## 2. The Unfolded Protein Response, ER Stress and Cancer

The ER is a specialized organelle that is responsible for the synthesis, assembly, folding, transport and degradation of a large number of membrane and secreted proteins [6]. The quality and fidelity of all of these steps is constantly monitored by the cell. The accumulation of unfolded or misfolded proteins in the ER lumen rapidly results in ER stress and activates the UPR process. The UPR combines several systems to slow down ongoing protein synthesis and to increase the folding capacity of the ER. If this reaction is successful, cellular protein synthesis resumes, and cellular homeostasis will be restored, facilitating survival of the ER stress condition. If ER stress persists, the UPR pathways will eventually induce oxidative stress and cell death [6]. Tumor cells have not only acquired the capacity to suppress death-inducing pathways, to induce angiogenesis and to reprogram their metabolism, as reviewed elsewhere [7], but also show an increased demand for protein synthesis and folding capacity. These demands favor an increased supply of nutrients and eventually, shape the tumor microenvironment, including the activities of invading immune cells. It is therefore not surprising that akin to NF-κB, ER stress has also been related to cancer, and this has been comprehensively reviewed recently [8,9,10]. NF-κB induction by the UPR does not only occur in highly proliferative tumor cells, but also in other pathophysiological situations, such as infection by viruses, as demonstrated for the hepatitis C virus or for human coronavirus 229E [11,12]. As discussed below, both the ER/UPR and NF-κB pathways operate through gene-regulatory mechanisms, ultimately inducing and fine-tuning the mRNA and protein expression of specific sets of genes. However, our knowledge on the interactions of the two systems and the levels and specificities of this type of crosstalk for certain types of cancer, for inflammatory and immune reactions or for infections is far from complete and represents an emerging area of investigation [13,14,15,16,17].

## 3. The ER Stress Sensors

ER stress is recognized by three sensors that are inserted into the ER membrane: protein kinase R (PKR)-like ER kinase (PERK), inositol-requiring protein 1α (IRE1α) and ATF6α (also called cyclic AMP-dependent transcription factor 6α). PERK and IRE1α share similar lumenal parts and possess cytosolic ser/thr kinase domains [18]. In non-stressed cells, the major ER chaperone binding-immunoglobulin protein (BIP), also called 78 kDa glucose-regulated protein (GPR78), binds to the ER-oriented parts of PERK and IRE1α and keeps them in a monomeric inactive state. Increased binding of BIP/GRP78 to misfolded proteins relieves both PERK and IRE1α and facilitates activation by dimerization (or oligomerization) followed by trans(auto)phosphorylation [19,20]. Active PERK then phosphorylates the eukaryotic translation initiation factor 2 (eIF2) subunit α to shut down translation but also activates the ATF4-dependent transcription program (see below). Phosphorylated IRE1α activates its own RNAase domain to catalyze the excision of 26 nucleotides of XBP1 mRNA, thereby generating spliced XBP1 mRNA. This transcript is translated into the active XBP1 protein, a multifunctional transcriptional regulator. ATF6α is a transcription factor that is processed into its mature form (ATF6f) by Golgi-associated proteases upon relief from BIP/GRP78 interactions. The released cytoplasmic part contains the basic-region leucine zipper (bZIP) transactivation domain. The three branches of the UPR often act in concert but can also be activated sequentially and with different strengths, allowing a multitude of outcomes spanning from the compensation of ER stress and the restoration of proteostasis to cell death as the ultimate effect [5]. As the mechanistic evidence for a connection of ATF6α/ATF6f and XBP1s to NF-κB is scarce, in this review, we concentrate on the crosstalk of PERK and IRE1α with the NF-κB system.

## 4. Regulation of eIF2-Dependent Translation Initiation by Phosphorylation

During de novo protein synthesis, a central event in translation initiation involves the assembly of a ternary complex composed of the multi subunit eIF2 complex loaded with both GTP and the initiator Met-tRNA with the ribosome at the start codon [21,22]. During translation initiation, eIF2-GTP is hydrolyzed, releasing eIF2-GDP from the ribosome. The recycling of eIF2-GDP to the GTP-bound form requires the guanine nucleotide exchange factor, eIF2β. Phosphorylation of the eIF2 subunit, eIF2α, by eIF2α kinases at ser51 converts the eIF2 initiation factor from a substrate to an inhibitor of eIF2β. The resulting drop in eIF2-GTP levels then suppresses general translation [21]. Phosphorylation of eIF2α is mediated by one of the four kinases: (i) heme-regulated inhibitor kinase (HRI, encoded by the gene *EIF2AK1*), (ii) RNA-activated protein kinase (PKR, encoded by the gene *EIF2AK2*), (iii) PERK (encoded by the gene *EIF2AK3*) and (iv) general control non-derepressible-2 (GCN2, encoded by the gene *EIF2AK4*). Phosphorylation of eIF2α is reversed by inducible growth arrest and DNA damage-inducible protein GADD34 (encoded by *PPP1R15a*) that targets protein phosphatase 1 (PP1) to dephosphorylate and inactivate eIF2α. PP1 activity is also under control of the constitutive repressor of eIF2α phosphorylation, CReP (encoded by *PPP1R15b*) [23,24,25,26]. The four eIF2α kinases are typically activated by infection (double stranded RNA, PKR), amino acid starvation (GCN2), heme-depletion (HRI) or by unfolded proteins in the ER (PERK). Despite their stimulus and cell type selectivity, they cooperate to mediate the phosphorylation of eIF2α, and mouse embryonic fibroblasts (Mefs) lacking PERK/GCN2/PKR have strongest reduction in phospho-eIF2α [27]. Notably, the eIF2 phosphorylation/dephosphorylation cycle is often disrupted in cancer to further promote or suppress translation, as comprehensively reviewed in [28].

## 5. Small Molecule Effectors as Prevailing Tools to Model ER Stress

A considerable number of reports addressing the mechanisms of ER stress have used chemical compounds to induce the UPR, in particular tunicamycin, thapsigargin, dithiotreitol (DTT), proteasome inhibitors and brefeldin A [6]. Tunicamycin inhibits ER-associated glycoprotein synthesis [29,30], while thapsigargin, a sesquiterpene lactone isolated from the plant, *Thapsia garganica* L., has long been known to potently inhibit the sarco-endoplasmic reticulum Ca^2+^-ATPase (SERCA) [31]. Thereby, thapsigargin depletes Ca^2+^ from the ER and is highly cytotoxic [32]. This has led to the testing of thapsigargin as an anti-cancer agent [31]. Another UPR-inducing compound is DTT, a reducing agent that disrupts disulfide bonds and thus, results in the accumulation of unfolded proteins [33]. In this review, we will specifically refer to mechanistic conclusions derived from such approaches using chemical effectors that allow exact and reproducible control of the experimental conditions, as opposed to alternative, more physiological settings

## 6. Evidence for Activation of the Canonical NF-κB Pathway through Phosphorylation of eIF2α

In 1995, using electrophoretic mobility shift assays (EMSA) which assess the in vitro DNA-binding activity of NF-κB proteins in nuclear extracts, Pahl and Baeuerle discovered that thapsigargin activates p65/p50 NF-κB subunits [34,35]. In 2003, Jiang et al. reported that this effect was abolished in Mefs lacking PERK or expressing an eIF2α S51A mutant [36]. The same effect was seen in thapsigargin-treated Mefs subjected to leucine starvation or exposed to UV light. In this case, NF-κB activation was reduced in cells lacking GCN2, the eIF2α kinase that functions as a sensor for amino acid starvation [36,37]. PERK was also shown to be required for activation of a luciferase reporter gene driven by NF-κB binding sites, demonstrating that this pathway increases NF-κB-dependent transcriptional activity in intact cells [36]. These authors did not observe inducible phosphorylation of IκBα or its degradation in response to thapsigargin. Rather, they found evidence that thapsigargin favors the release of p65/p50 from cytosolic IκBα complexes, an effect that was absent in Mefs expressing the eIF2α S51A mutant. The phospho-eIF2α-dependent regulation of p65 by thapsigargin was confirmed by Deng and coworkers [38]. This group also designed experiments to investigate the role of eIF2α phosphorylation independent from stress signals. For this, they constructed a protein consisting of the cytoplasmic PERK kinase domain fused to a protein module that allowed conditional tethering (or oligomerization) of the PERK domains by a bivalent cell permeable compound. Indeed, the addition of the cross linker to stable cell lines induced phosphorylation of eIF2α, and nuclear translocation of p65 and induction of its transcriptional activity [38]. However, in marked contrast to Jiang et al., this was paralleled by partial degradation of IκBα. The authors concluded that the PERK–phospho-eIF2α pathway inhibits the synthesis of IκBα but does not affect the pre-existing protein, as revealed by pulse chase experiments that monitored de novo protein synthesis [38]. Thus, while both studies showed that the ER stress pathway activates NF-κB, they disagreed on the underlying cytosolic activation mechanism. In both of these studies, no evidence for the phosphorylation of IκBα or for activation of IκB kinases (IKKs) was found. Additionally, UV treatment reduces cellular IκBα protein levels, an effect that is absent in eIF2α S51A Mefs and thus, critically requires eIF2α phosphorylation [39]. UV treatment globally shuts down translation through the PERK–eIF2α pathway suggesting that this is the key mechanism for IκBα decay [40]. However, UV treatment was later shown to also induce degradation of IκBα through C-terminal phosphorylation of the PEST domain by casein kinase II (CK2) providing an alternative explanation for IκBα depletion [41]. To date, the paradigm of ER stress-mediated suppression of constitutive IκBα protein synthesis is still suggested to be the prevailing mechanism of ER–NF-κB crosstalk and this view has been emphasized in several reviews [13,14,15,42,43].

## 7. Regulation of IκBα Half-Life: The Major Control Point Affected by ER Stress?

The regulation of IκB protein levels is key to NF-κB activation, as also shown by the early observation that the inhibition of protein synthesis by cycloheximide (CHX) is sufficient for NF-κB activation [44]. This raises the question of what is known about the half-life of IκBα in diverse conditions and whether these observations match the conclusion that ER stress mainly activates NF-κB indirectly by suppressing constitutive IκBα steady state levels. Amongst others, the IκBα protein (and its related family members, IκBβ and IκBε) can be regarded as one of the most powerful and universal negative regulators of NF-κB [45]. A plethora of infectious and inflammatory conditions, including cytokines such as interleukin(IL)-1 or tumor necrosis factor(TNF)α trigger the active and rapid destruction of IκBα by the well-characterized phosphorylation-dependent proteasomal degradation pathway [46,47]. This allows NF-κB subunits to translocate to the nucleus and to induce transcription of numerous target genes, including the *NFKBIA* gene that encodes IκBα [48]. The newly synthesized IκBα sequesters NF-κB from promoters and enhancers, retaining it in the cytoplasm [49]. This major negative feedback loop shuts down NF-κB transcriptional activation in multiple cell types [45,50,51]. However, with respect to the regulation of basal IκBα protein levels, several studies have reported different half-lives in a number of cell types and suggested different ways in which this might be regulated [52,53,54,55]. Mathes et al. provided one of the most comprehensive studies on the regulation of basal IκBα levels. By studying genetically modified Mefs, they concluded that cells essentially contain two pools of IκBα, a small fraction of free IκBα (around 15% of total) and IκBα bound to NF-κB. The level of free IκBα is severely reduced in cells lacking the p105, c-REL and p65 (RELA) subunits, showing that the NF-κB subunits are required for protein stabilization of IκBα. They may also be required for basal transcription of the *NFKBIA* gene, an issue not addressed in this study. The half-life of free IκBα ranges from 10 min to 20 min, whereas that of the NF-κB-bound IκBα is more stable, ranging from 8 h to 10 h. Free IκBα is degraded by the proteasome through IKK- and ubiquitination-independent events involving the C-terminal PEST domain [56]. Mathes and coworkers also showed that the free IκBα degradation pathway allows for fast and maximal activation of NF-κB, as cells expressing a degradation-resistant C-terminal IκBα mutant (ΔC288) have a delayed and dampened TNFα-inducible NF-κB activation profile [56]. It follows that our understanding of the true role of the PERK–eIF2α pathway in the regulation of the IκBα–NF-κB complex is still hampered by a lack of data concerning a detailed analysis of the effects of ER stress on IκBα protein stability and the stoichiometry of free versus bound IκBα in physiological and pathophysiological situations, independent from highly toxic chemicals, such as thapsigargin.

## 8. Additional Levels of Cytosolic NF-κB Regulation by ER Stress

**Figure 1:**
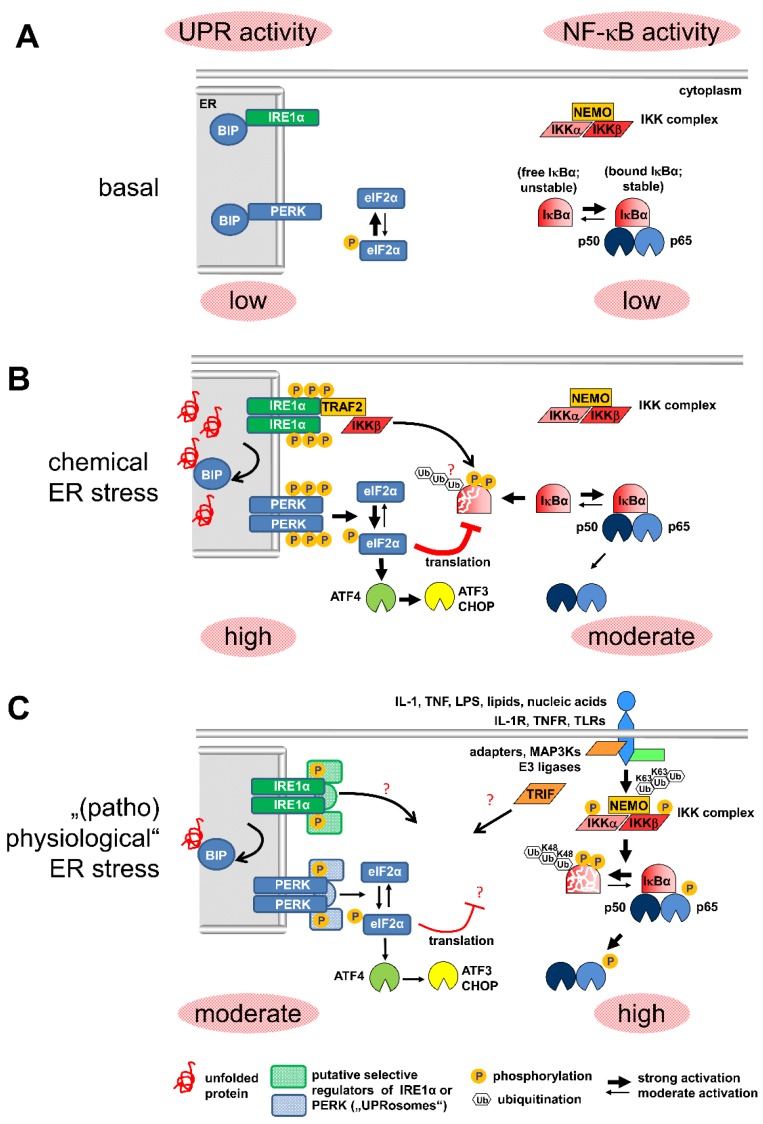
Integration of unfolded protein response (UPR) and NF-κB signaling. (**A**) In the absence of signals, most cells have no or very low basal activity of the UPR and of the canonical NF-κB pathway. The protein kinases IRE1α and PERK are kept inactive by binding to the chaperone BIP/GRP78 within the endoplasmic reticulum (ER) lumen. Most of the IκBα inhibitor is bound to NF-κB subunits retaining them in the cytoplasm. (**B**) Different classes of chemical stressors (tunicamcyin, thapsigargin or dithiotreitol (DTT)) increase the unfolded protein load in the ER causing massive auto-phosphorylation and activation of both IRE1α and PERK. IRE1α binds to the adapter protein TRAF2 and (indirectly) to the protein kinase IKKβ. PERK phosphorylates the eukaryotic translation initiation factor eIF2α causing translational shut-off for multiple proteins including the free unbound IκBα. Destruction of free IκBα also requires phosphorylation by IRE1α-associated IKKβ. (**C**) During infection, inflammation or cancer, strong activation of NF-κB by cytokines or toll-like receptor (TLR) agonists occurs in parallel to ER stress. In this case, the formation of specific signaling complexes at PERK and IRE1α sensors by still putative “UPRosomes” is suggested to restrict maximal ER stress, thereby contributing to context-specific gene activation or repression.

Emerging evidence has shown that ER stress also modulates (or requires) critical upstream regulators of the NF-κB pathway. Thapsigargin or tunicamycin can also induce NF-κB through the catalytic activity of the IRE1α kinase. In this case, IRE forms a complex with IκB kinase (IKK)β and tumor necrosis factor receptor (TNFR)-associated factor (TRAF)2. In this model, thapsigargin-induced cell death involves the strongly increased synthesis and secretion of TNFα which is blocked in cells expressing a non-degradable IκBα mutant or in cells lacking IRE1α [57,58]. TRAF2 is an ubiquitous adaptor protein of TNFα and toll-like receptor (TLR) pathways [59]. Another TLR adaptor protein called toll-interleukin-1 receptor domain-containing adapter protein inducing interferon β TRIF or TIR domain-containing adapter molecule 1 (TCAM-1) is essential for activation of the lipopolysaccharide (LPS) target gene, IL-1β. Pretreatment of macrophages with thapsigargin or tunicamycin strongly increases LPS-triggered synthesis, processing and release of mature IL-1β in a TRIF-dependent manner [60]. However, when macrophages are pretreated with low doses of LPS, TRIF specifically mediates the suppression of tunicamycin-induced CHOP and ATF4 expression [61,62]. These data reveal that complex positive or negative crosstalk loops between the UPR and NF-κB also operate through secreted cytokines (TNFα, IL-1β) or TLR agonists (LPS) that are all well known for strongly activating the canonical NF-κB pathway [63].The IRE1α-dependent mechanism seems to cooperate with the PERK pathway for regulating NF-κB. DTT- or thapsigargin-induced NF-κB activity (as determined by EMSA) does not depend on IKKα, but rather on IKKβ, as revealed by the analysis of Mefs lacking IKKβ or IKKα/β or re-expressing a kinase-inactive mutant of IKKβ. Thapsigargin-mediated IκBα decay is abrogated in cells reconstituted with an IκBα super repressor mutant (SS32/36AA), suggesting that IκBα decay depends on IκBα phosphorylation. Puzzling though, neither IκBα phosphorylation, nor IKK activity, were directly triggered by thapsigargin or DTT through PERK. Rather, NF-κB activity and IκBα degradation were reduced in IRE1α-deficient cells. The half-life of IκBα is around 5 h in wild type cells but IκBα is very stable in IRE1α -/- cells. Both, reduced basal phosphorylation of IκBα and diminished NF-κB activity in IRE1α-deficient cells were restored by IRE1α, but also by IKKβ (but not IKKα) [64]. These results suggest that basal IKK activity, maintained by IRE1α, is critical for the activation of NF-κB when PERK-induced translation inhibition (by thapsigargin) occurs. Tam et al. also showed that the extent of DTT- or thapsigargin-induced activation of NF-κB correlates with the amount of IRE1 re-expressed in IRE1α-deficient cells. NF-κB activity inversely correlates with CHX-mediated translational inhibition, showing a proportional correlation with the level of NF-κB activation. Based on these genetic experiments, IRE1α-dependent regulation of basal IKK activity is necessary for effective activation of NF-κB by PERK [64]. Such a link may also operate in disease, as demonstrated in a model of dextran sulfate-induced colitis in mice, where IKKα suppressed ER stress through the TNFR and nucleotide-binding oligomerization domain-containing protein (NOD)1/2 receptor-mediated pathways. Mice expressing a non-activatable IKKα mutant show increased IRE1α-dependent ER stress [65]. Thus, these data show that upon chemical ER stress, the PERK and IRE1α branches of the UPR may, in fact, work in concert to modulate the NF-κB pathway. However, it remains an open question how exactly IRE1α regulates basal or inducible IKK activity and vice versa. In conclusion, multiple lines of evidence suggest that strong activation of the canonical NF-κB pathway (by IL-1, TNFα, LPS) is accompanied by (moderate) activation of the IRE1α and PERK branches of the UPR. The various levels of interplay between NF-κB and the UPR are schematically displayed in Figure 1.

## 9. Cross-Interference of ER Stress and NF-κB at the Level of Transcription Factors and Gene Regulation

Arguably the most striking but least discussed aspect of ER stress interactions with the NF-κB system involves the chromatin response within the nucleus. Early studies showed that thapsigargin strongly induces the protein levels of the transcription factors ATF3 and ATF4 and CHOP (also called growth arrest and DNA damage-inducible protein (GADD)153 or DNA damage-inducible transcript 3 protein (DDIT3)). Besides transcriptional induction, this occurs by a unique mechanism that allows preferential translation from downstream open reading frames (ORFs) of ATF4 or CHOP (and also GADD34) [66,67,68]. ATF3 seems to be mainly regulated by transcriptional mechanisms involving ATF4, CHOP and Jun family members [69,70]. ATFs belong to the bZIP family of TFs, while CHOP is a member of the C/EBP family of TFs. Thapsigargin strongly induces the expression of all three of them [27]. This effect is absent in PERK-deficient cells, while during amino acid starvation, GCN2 is employed for their expression. Importantly, cells lacking ATF4 do not express ATF3 or CHOP, whereas cells lacking ATF3 normally induce ATF4 and CHOP. ATF4 and ATF3 are also required for the regulation of GADD34, thus forming a negative feedback loop for eIF2α phosphorylation. These landmark studies defined the PERK/GCN2–ATF4–ATF3–CHOP pathway and clearly established a hierarchy of the three TFs within this signaling cascade [27,71]. Upon amino acid starvation, ATF3 and ATF4 bind to the amino acid response elements (AARE) of the ATF3 promoter and numerous metabolic genes, such asparagine synthetase (ASNS), sodium-coupled neutral amino acid transporter-2 (SNAT2), and the γ-glutamyl cyclotransferase (CHAC1) but also to the vascular endothelial growth factor (VEGF) promoter [72,73,74,75]. Interestingly, some of the genes regulated by nutritional stress are well known inflammatory NF-κB target genes, such as *IL8* and *CXCL2* [74,76,77,78]. Thus ATF4 and ATF3, together with the NF-κB subunits, form a transcription factor network that coordinates metabolic gene expression programs during nutritional and ER stress [79]. As nutritional and metabolic changes are major hallmarks of cancer, these observations form a natural link between ER stress, the UPR and the NF-κB system in malignant disease that warrants further investigation.

Less is known about the regulation of ER stress target genes by NF-κB subunits. In one report, the thapsigargin-inducible expression of CHOP and ATF4 was unchanged in cells lacking p65 [36]. Expression of BIP/GRP78 upon thapsigargin treatment is normal in PERK-deficient cells suggesting that this crucial ER chaperone is regulated independently from ATF3, ATF4 or CHOP [27]. Interestingly, another observation from the study of Tam and coworkers is that thapsigargin-induced expression of BIP/GRP78 is reduced in p65-deficient Mefs, and p65 binds to the BIP/GRP78 promoter, suggesting a new direct link from NF-κB subunits to ER stress target genes [64].

The connection of ATF3/ATF4/CHOP to the NF-κB system also became apparent with the advent of bioinformatics approaches and genome-wide assays correlating DNA-binding profiles (obtained by chromatin immunoprecipitation sequencing (ChIP-seq)) with mRNA expression. In macrophages, a combination of microarray analyses and motif searches resulted in the identification of ATF3 as an early LPS-induced gene that bound in the proximity of p50 (REL) NF-κB sites. ChIP-qPCR confirmed the binding of NF-κB and ATF3 to the *IL6* and *IL12b* (*IL12p40*) promoters [80]. ATF3 is required for the recruitment of HDAC1 and negative regulation of eleven LPS-target genes [80]. This was confirmed by a systems biology approach showing that ATF3 attenuated LPS-induced *IL6* as part of a regulatory circuit that involves sequential activation and cooperative activity of NF-κB and C/EBPδ. Depletion of ATF3 or C/EBPδ or pharmacological inhibition of NF-κB by the IKK inhibitor SC-514 disrupted the kinetics and amplitude of LPS-induced *IL6* expression [81]. ATF3 was also identified as a high-density lipoprotein-inducible repressor of TLR-induced proinflammatory cytokines. This first ATF3 ChIP-seq study confirmed the inducible binding of ATF3 to the promoters of the *IL6*, *IL12p40* and *TNFA* genes [82]. ATF3 is also a type I interferon-inducible negative regulator of multiple interferon response genes (ISGs) and binds to the *IFNβ* promoter [83]. These studies assigned a broad and largely negative regulatory role to ATF3 in regulating the NF-κB response and provided links to immunity and cancer (also reviewed in [84].

**Figure 2:**
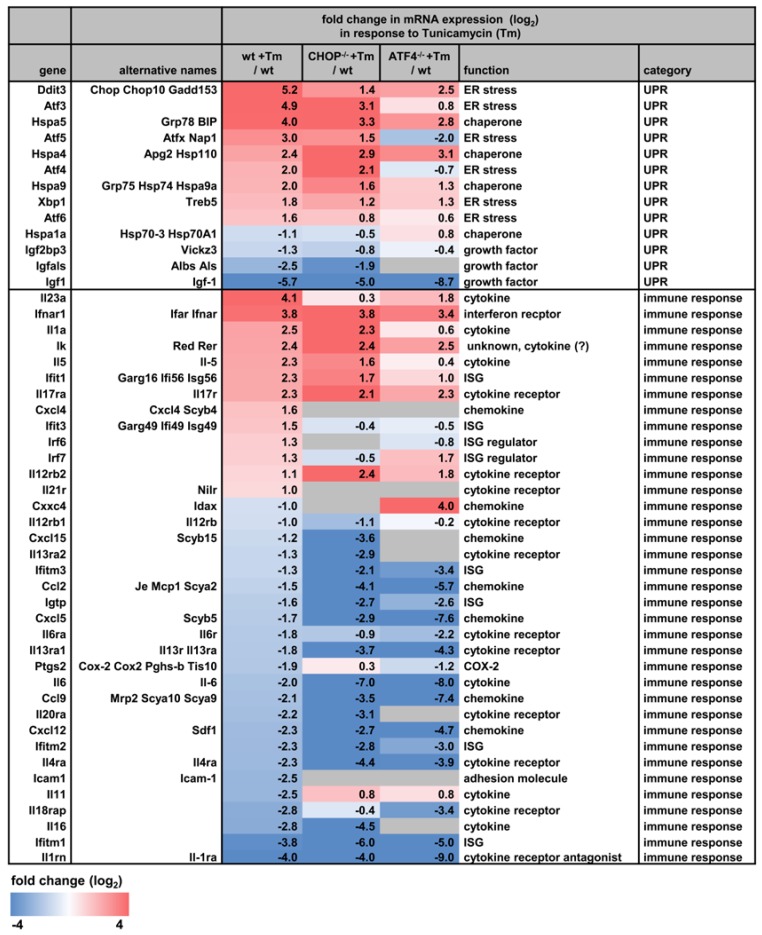
ATF4- or CHOP-dependent regulation of the UPR and of immune modulators by chemical ER stress. Published RNA-seq data sets from wild type murine embryonic fibroblasts or cells deficient in CHOP (CHOP-/-) or ATF4 (ATF4-/-) treated with tunicamycin (Tm) for 10 h or left untreated were extracted from GEO (GSE35681). Data were filtered for genes regulated by at least two-fold. The gene list is sorted by fold change compared to untreated wild type cells. Shown is a selection of prototypical target genes of the UPR and of immune responses. Gray colored boxes indicate the absence of expression.

Han et al. systematically identified the target genes of ATF4 and CHOP in response to tunicamyin by a combination of ChIP-seq and RNA-seq approaches using ATF4 or CHOP-deficient Mefs. They showed that ATF4 and CHOP co-occupy many genomic regions and identified around 3000 binding sites within the genome. This analysis also revealed several hundred common, but also unique, target genes of both factors. Upregulated gene sets were clearly enriched for ER stress target genes involved in protein folding, amino acid synthesis and protein transport, while downregulated genes were involved in proliferation, wound healing, anti-apoptosis, and steroid and lipid synthesis [85]. We interrogated this data set for factors that may represent the crosstalk of ER stress with (NF-κB-dependent) immunoregulatory genes. Indeed, as illustrated by the selection shown in Figure 2, tunicamycin also regulates multiple genes with annotated functions in immune responses such as various cytokines and their receptors (e.g., *Il23a*, *Il1a*, *Il6*, *Ifnar1*, *Il17ra*, *Il6ra*), chemokines (*Ccl2*, *Ccl9*), adhesion molecules (*Icam1*) and prostaglandin synthetases (*Ptgs2*, Cox-2). In many cases these are suppressed in an ATF4- or CHOP-dependent manner, while *Il23a* is induced as previously reported (Figure 2), [86,87].

**Figure 3:**
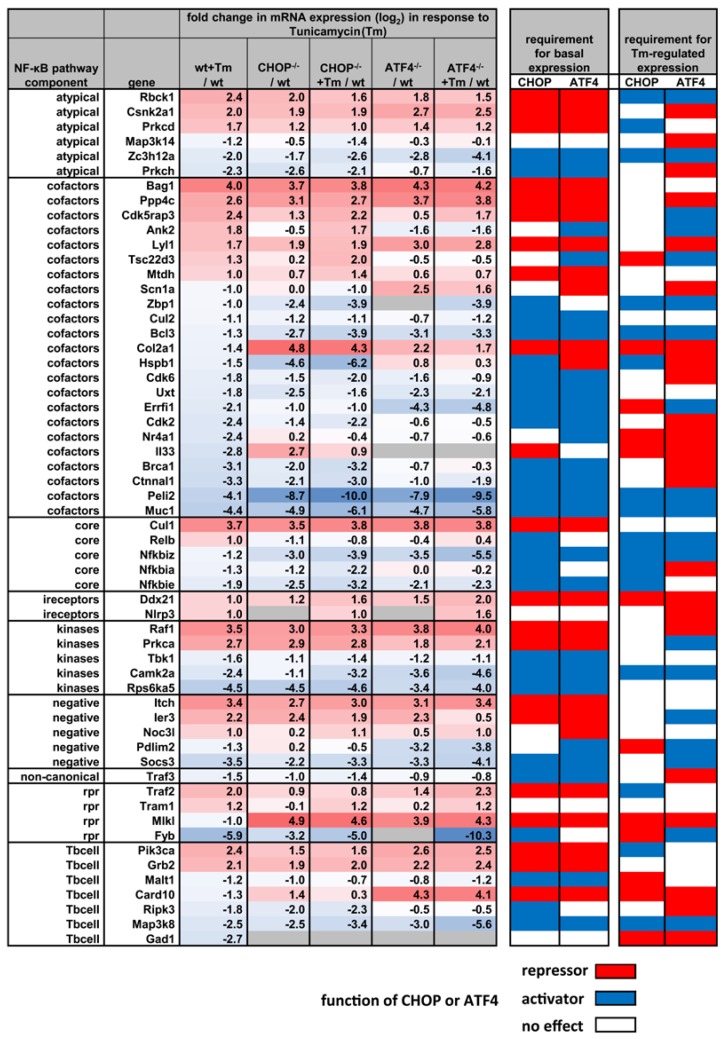
ATF4- or CHOP-dependent modulation of NF-κB regulators by chemical ER stress. The same data sets described in Figure 2 (GSE35681) were filtered for mRNA expression of 262 genes with a documented role in the regulation of NF-κB in diverse systems (as revealed by searching public data bases). The left column shows their role in regulation of the canonical (core), non-canonical, or atypical NF-κB pathways and some additional information on their established functions according to (Perkins, 2007). Two hundred and thirty-two of these factors were found to be expressed in all conditions. The left heatmap summarizes 58 components (25% of all components) that were deregulated by at least two-fold by tunicamycin (Tm) in wild type cells and the corresponding changes in CHOP- or ATF4-deficient Mef cells. The right color map categorizes the effects of loss of CHOP or ATF on basal and Tm-inducible expression of the NF-κB components based on log_2_ differences of more than 0.5. Gray colored boxes indicate the absence of expression. Abbreviations are as follows: ireceptors, intracellular receptors; rpr, receptor-proximal component; Tbcell; NF-κB pathway components mainly characterized in T- or B-lymphocytes.

The significant overlap in target genes of NF-κB and the UPR transcription factors, ATF4- and CHOP, was also seen by the comparative analysis of gene expression sets visualized in Figure 3. These data show ATF4- and CHOP-dependent regulation of 58 genes that have a documented role in regulation of the NF-κB signaling pathway, including three IκBs, (IκBα, IκBe, IκBz). Data sets of this kind, therefore, not only highlight how closely ER stress and the NF-κB response are intertwined, they also provide a rich resource for further analyses of the exact mechanisms of crosstalk between these two systems. In physiological settings, this crosstalk between ER stress transcription factors is not necessarily always negative or repressive. For example, it was recently shown that ATF4 is a positive regulator for LPS-induced *Ccl2* expression in the endothelium and mediates leukocyte infiltration within the retina, while ATF3 can support breast cancer metastasis [88,89]. *Ccl2* is a prototypical NF-κB target gene that also requires additional transcription factors from the c-Jun/Fos family for full activation [90]. Likewise, all three canonical ER stress TFs, ATF4, ATF3 and CHOP, engage in multiple further interactions with other TFs that also shape their influence on the NF-κB response, as reported in several reviews [91,92]). More generally, the main ER stress TFs are likely always integrated into large protein–protein interaction (PPI) networks and co-occupy their target genes, as shown recently by large studies that combined multiple ChIP-seq and mass spectrometry profiling approaches [93,94].

## 10. The Impact of Pharmacological Inhibition of PERK or IRE1α on NF-κB Function

As outlined above, much of the mechanistic knowledge on the ER stress–NF-κB relationship has been derived from genetically altered mouse fibroblasts. Recently, fast acting pharmacological compounds have become available, facilitating dissection of ER stress pathways in more complex models. In 2012, a novel, small molecule, ATP-competitive PERK inhibitor (GSK2606414) was reported to inhibit thapsigargin-induced PERK autophosphorylation in intact cells within the nM range. This inhibitor is highly selective and only affected 20 out of 294 kinases tested by >85% at 10 µM and suppressed tumor growth in a mouse xenograft model [95]. An optimized version of this compound (GSK2656157) was 1000-fold more active on PERK against a panel of 300 kinases, including HRI, PKR and GCN2 [96,97]. Again, this compound suppressed a range of human xenograft tumors and inhibited PERK autophosphorylation, eIF2α phosphorylation and the induction of UPR target genes in cell lines and tumors [96]. However, the inhibitor was also cytotoxic to the exocrine/endocrine pancreas tissues in non-tumor controls consistent with the results from PERK-deficient mice and loss of function PERK mutations in humans [98,99]. These and other data suggest tissue-specific functions of PERK in the development and exocrine function of the pancreas that may also partly operate through P-eIF2α independent effects [100]. The latter is supported by the observation that the PERK inhibitor caused cell death in human HT1080 tumor cells engineered to express the eIF2α S51A mutant, thus bypassing the P-eIF2α pathway [101]. Like for any protein kinase inhibitor, this may simply be the result of off-target effects and indeed, there is a single study suggesting that both PERK inhibitors also inhibit RIPK1 in the TNFα pathway [102]. However, recent studies have reported suppressive effects of PERK inhibitors on prototypical NF-κB target genes in diverse models. Using transcriptome analyses, Iwasaki et al. found that saturated fatty acids acting as proinflammatory factors induce nuclear translocation of ATF4 and p65 in macrophages. As demonstrated by ChIP, both TFs are co-recruited to the *IL6* promoter and activate *IL6* transcription. A functional interaction between p65 and ATF4 was found by reduced nuclear translocation and promoter binding of p65 in ATF4-deficient cells [103]. Iwasaki et al. also showed that the expression of ATF4 was suppressed by the PERK inhibitor, GSK2656157. These data confirm, in physiological settings, that ATF4 links various metabolic stresses to the NF-κB chromatin response [103]. In a rat model of intervertebral disc degeneration, silencing of PERK or ATF4 or application of GSK2606414 also suppressed inducible *Il6* expression in addition to *Tnfa* [104].

A further inhibitor acting on the PERK–eIF2α pathway is salubrinal. This compound suppressed eIF2α dephosphorylation through the GADD34/PP1 and CReP/PP1 complexes [105]. Recently, salubrinal was shown to inhibit TNFα (but not IL-1)-induced activation of NF-κB and the expression of *Ccl2*/*Mcp-1*. This effect was not abrogated in cells with siRNA-mediated suppression of eIF2α, suggesting that the salubrinal effect occurred through PP1, independent from eIF2α. In support of this, the same effect was seen with guanabenz, another PP1 inhibitor [106].

Another newly developed inhibitor is based on the finding that oligomerization of the IRE1α kinase domain controls the catalytic activity of the adjacent endoribonulease domain. Ghosh et al. developed an optimized small molecule inhibitor called kinase-inhibiting RNase-attenuator (KIRA)6. KIRAs disrupt IRE1α oligomerization thereby suppressing the RNase activities of IRE1α [107]. IRE1α is often mutated in cancer and it will be interesting to learn if KIRA compounds also modulate NF-κB activities in malignant diseases [9,107]. Consistent with the observations cited above, Keestra et al. found that thapsigargin induces *IL6* mRNA and protein levels through NOD1/NOD2 receptors—two cytosolic sensors of bacterial peptidoglycans. Interestingly, IL-6 secretion was suppressed by KIRA6 but not by GSK2656157 [108]. Together, these pharmacological approaches provide independent support for a link between ER stress and NF-κB, although the underlying mechanisms and the specificity of the effects require further investigation.

## 11. Concluding Remarks and Open Questions

**Figure 4:**
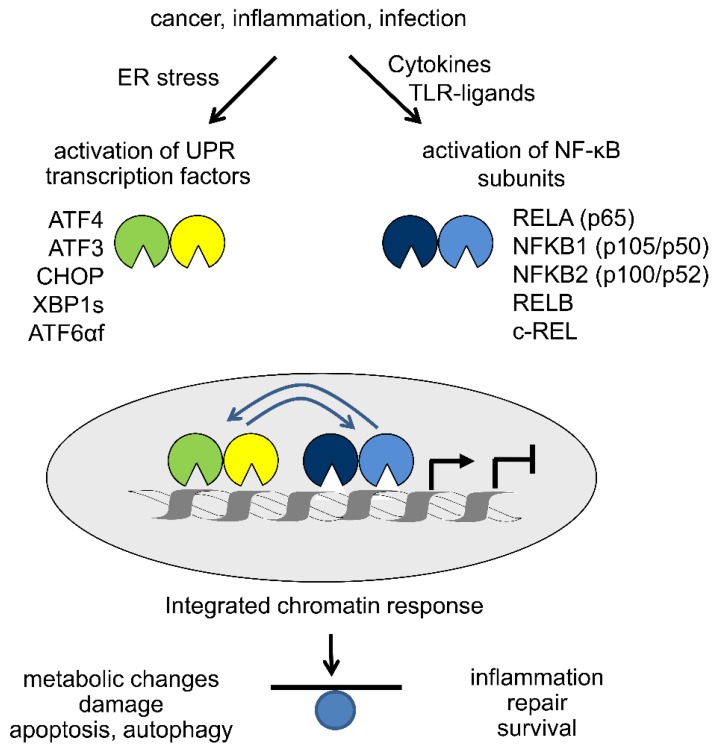
Integration of UPR and NF-κB signaling at chromatin. Despite representing unique signaling cascades, new data indicate that UPR and NF-κB pathways converge within the nucleus through ten major transcription factors. The combinatorial occupancy of numerous genomic regions (enhancers and promoters) coordinates the transcriptional activation or repression of hundreds of genes that collectively determine the balance between metabolic and inflammatory phenotypes and the extent of apoptosis and autophagy or repair of cell damage and survival.

In this review we have summarized studies that connect the ER stress-mediated activation of the UPR to activation of the NF-κB pathway. So far, the evidence has been mainly derived from genetic experiments and chemical activators of ER stress, with a focus on cytosolic signaling pathways. However, as schematically depicted in Figure 4 these two pathways can also be viewed as a combinatorial system of ten transcription factors that cooperate to shape gene activation patterns and protein folding capacities. In the future, it will be important to test the concept of specific signaling complexes assembling at IRE1α and PERK during infection, inflammation and in malignant processes. The idea of such “UPRosomes” is attractive as it provides ample opportunity to adapt the extent and kinetics of UPR activation to the activity of other signaling pathways, such as NF-κB [5]. Another important area of research will be the systematic investigation of combined UPR and NF-κB activation on transcriptional programs and chromatin structure in disease.
